# Detection of circulating Tg-mRNA in the follow-up of papillary and follicular thyroid cancer: how useful is it?

**DOI:** 10.1038/sj.bjc.6601991

**Published:** 2004-06-22

**Authors:** F A Verburg, C J M Lips, E G W M Lentjes, J M H de Klerk

**Affiliations:** 1 Department of Nuclear Medicine, University Medical Center Utrecht, Heidelberglaan 100, 3584 CX Utrecht, The Netherlands; 2 Department of Endocrinology, University Medical Center Utrecht, Heidelberglaan 100, 3584 CX Utrecht, The Netherlands; 3 Department of Laboratory Medicine, University Medical Center Utrecht, Heidelberglaan 100, 3584 CX Utrecht, The Netherlands

**Keywords:** thyroglobulin, Tg-mRNA, thyroid carcinoma, RT–PCR, gene profiling

## Abstract

To investigate the usefulness of thyroglobulin mRNA (Tg-mRNA) detection in peripheral blood in the follow-up of papillary and follicular (differentiated) thyroid cancer, a literature study was performed. Both evidence for and evidence against the usefulness of Tg-mRNA detection were found. Also, evidence for the expression of Tg-mRNA in cells other than normal or neoplastic thyroid follicular cells was found. It is concluded that currently Tg-mRNA detection is not a useful tool in the follow-up of differentiated thyroid carcinoma, but that the concept of using RT–PCR measurements during follow-up still warrants further research.

Differentiated thyroid cancer is among the most manageable of cancers; with a 10-year survival of 80–95% (Tubiana *et al*, 1985), its prognosis is good. However, patients with differentiated thyroid cancer can never be considered ‘cured’; recurrences do occur, sometimes as late as 30 years after the original diagnosis (Tubiana *et al*, 1985; Mazzaferi and Jhiang, 1994). It is therefore imperative to regularly check these patients during a life-long follow-up.

Checking patients for the presence of persistent or recurrent disease can be carried out by regular measurement of serum thyroglobulin (Tg) levels, both during suppressed TSH and during stimulation with high TSH levels, achieved either through levothyroxine (LT4) withdrawal or administration of recombinant human TSH, and periodic radioiodine whole-body scintigraphy (WBS) after withdrawing LT4 supplementation.

However, both these methods do have drawbacks. It often happens that I-131 WBS is negative, but serum Tg is readily detectable (Pineda *et al*, 1995; Pachucki and Burmeister, 1997; Fatourechi and Hay, 2000). One should remember that measurement of Tg levels cannot be considered reliable if antibodies against Tg are detected (Feldt-Rasmussen and Rasmussen, 1985; Mariotti *et al*, 1995; Spencer and Wang, 1995).

The limitations of current techniques have prompted research into new ways of detecting persistent or recurrent thyroid cancer. One of these techniques is detection of circulating thyroid cells by measurement of thyroglobulin messenger RNA (Tg-mRNA) in peripheral blood, first reported by Ditkoff *et al* (1996). This technique seems to be quite promising according to some authors (Ditkoff *et al*, 1996; Ringel *et al*, 1998, 1999; Biscolla *et al*, 2000; Savagner *et al*, 2002), but the results of other studies contradict its usefulness (Bojunga *et al*, 2000; Bugalho *et al*, 2001; Bellantone *et al*, 2001; Takano *et al*, 2001; ; Eszlinger *et al*, 2002; Span *et al*, 2003, Elisei *et al*, 2004).

The goal of this study is to review the pros and cons of Tg-mRNA detection in peripheral blood and determine whether it can currently be considered useful in the follow-up of differentiated thyroid cancer.

## THYROGLOBULIN MRNA

Thyroglobulin mRNA is a 8.7 kilobase (kb) transcript of the Tg gene, which covers at least 300 kb of DNA (Baas *et al*, 1986). It codes for Tg, a 660 kilodalton (kDa) glycoprotein that serves as a prohormone for thyroid hormone production.

With 2.6% of the total transcription products of thyrocytes, Tg-mRNA is the most highly expressed mRNA transcript in normal thyrocytes (Pauws *et al*, 2000). In neoplastic thyroid tissue, however, its expression is considerably lower (Ohta *et al*, 1991; Hoang-Vu *et al*, 1992; Lazar *et al*, 1999; Ringel *et al*, 2001).

## TECHNIQUE OF DETECTION OF TG-MRNA IN PERIPHERAL BLOOD

After a blood sample is drawn from a patient, total RNA is extracted from the sample, either directly from the blood or from the mononuclear layer after separation by using commercially available kits. The method varies only in details between the studies reviewed here.

Subsequently, an aliquot, usually 1 *μ*g, of total RNA is reverse-transcribed using various commercially available kits containing viral reverse transcriptase (RT). The cDNA acquired from this reaction is then amplified using a polymerase chain reaction (PCR). Quantification of these reactions can be performed with one of several methods.

The amplified DNA sample is then analysed Tg-mRNA content. This can be carried out using a gel electrophoresis of the sample with Tg-mRNA- negative and -positive controls, or by other methods, based on detection of a specific sequence of RNA.

Sensitivity of assays can vary: Bojunga *et al* (2000) reported using two different assays, one of which had a lower limit of detection of 50–100 Tg-mRNA-producing cells per ml blood and the other a lower limit of detection of 10–20 Tg-mRNA-producing cells per ml. Ringel *et al* report being able to detect 10 Tg-mRNA-producing cells per ml (Ringel *et al*, 1998).

## SIGNIFICANCE OF TG-MRNA DETECTION IN PERIPHERAL BLOOD

Investigation of the applicability of the RT–PCR reaction for the detection of Tg-mRNA was based on the assumption that patients with adequately treated thyroid cancer should not have circulating Tg-mRNA-producing cells, nor should individuals without thyroid cancer. Although the first studies showed promising results, later reports pointed to some problems with this technique and were sceptic concerning its usefulness.

Ditkoff *et al* (1996) first investigated the possibility of detecting circulating malignant thyroid cells using an RT–PCR reaction for the detection of Tg-mRNA; this could serve as an indicator of postoperatively present metastatic thyroid cancer.

This investigation was based on the assumption that patients with adequately treated thyroid cancer should not have circulating Tg-mRNA-producing cells, nor should individuals without thyroid cancer.

### Pro

Ditkoff *et al* (1996) were the first to investigate the possibility of detecting circulating malignant thyroid cells using RT–PCR. They found that Tg-mRNA could be detected in all nine patients with known metastatic thyroid cancer. Seven out of 78 patients with no currently known metastases, of whom five had a history of surgically treated metastases, also showed detectable Tg-mRNA in peripheral blood samples. No Tg-mRNA was detected in six patients with benign thyroid disorders or in seven healthy subjects. The study of Ditkoff *et al* therefore clearly demonstrated the usefulness of Tg-mRNA detection in the follow-up of thyroid cancer patients by having positive cases and only negative controls.

Two studies by Ringel *et al* (1998, 1999) showed that Tg-mRNA detection could be useful in the follow-up of differentiated thyroid cancer. In the first study, 33 patients had either thyroid bed uptake (*n*=19) or metastatic iodine-avid tissue (*n*=14) on the most recent withdrawal scan. In 12 out of 19 patients with thyroid remnants on the most recent follow-up scintigram, Tg-mRNA could be detected in their blood. In addition, all 14 patients with metastatic disease showing on the most recent follow-up scintigram had detectable Tg-mRNA in their peripheral blood. Serum Tg levels were detectable in only 12 of these 33 patients. Seven out of 35 patients with negative scintigrams were positive for Tg-mRNA too. All 10 healthy control subjects turned out to be positive for Tg-mRNA in peripheral blood due to the improved sensitivity of the RT–PCR.

In the second study of Ringel *et al*, a quantitative method of Tg-mRNA detection was used. Using a threshold of 3 pg Tg Eq *μ*g^−1^ thyroid RNA for the detection of Tg-mRNA, analysis was positive in 38% of patients with a negative follow-up scintigram, 75% of patients with thyroid bed uptake, 84% of patients with cervical/regional disease and 94% of patients with distant metastases. Thyroglobulin antibodies were shown not to affect the result of the analysis.

### Contra

Bojunga *et al* (2000) reported that using a ‘normal sensitivity’ (30 cycles of PCR) resulted in the detection of Tg-mRNA in nine out of 13 patients with thyroid cancer and known metastases, 63 out of 137 patients with a history of thyroid cancer without known metastases, 21 out of 85 patients with benign thyroid disorders and in nine out of 50 control subjects. Using a ‘high sensitivity’ (40 cycles of PCR), however, resulted in the detection of Tg-mRNA in peripheral blood of 11 out of 13 patients with thyroid cancer and known metastases, 111 out of 137 patients with a history of thyroid cancer without known metastases and also in 61 out of 85 patients with benign thyroid disorders and 41 out of 50 control subjects.

Takano *et al* (2001) reported in their study that Tg-mRNA could be detected in samples from all patients who have had a thyroidectomy. Additionally, no statistically significant difference in expression levels could be found between patients with and patients without metastases in a quantitative analysis.

### Illegitimate transcription of Tg-mRNA

Tissue specificity of Tg gene expression by detection of Tg-mRNA in various human tissues obtained through routine surgery was investigated by Bojunga *et al* (2000). Using a ‘normal sensitivity’, they found Tg-mRNA expression to be specific for thyroid tissue. Using ‘high sensitivity’, on the other hand, resulted in the detection of Tg-mRNA transcripts not only in thyroid tissue but also in various other tissues.

This confirms the findings of Tallini *et al* (1998), who, when studying 10 non-thyroid malignant human cell lines and 11 control subjects (including one patient who had had a total laringectomy for squamous cell carcinoma with a complete thyroidectomy), found no detectable expression of Tg-mRNA after 30 cycles of PCR, but found detectable Tg-mRNA expression in all samples after 40 cycles of PCR.

On top of this, Bugalho *et al* (2001) reported in a study of healthy individuals and patients who have had a thyroidectomy for reasons other than thyroid cancer that expression of Tg-mRNA was detectable in all subjects, and that quantitative analysis revealed no significant difference between those with and those without thyroid glands. Furthermore, they found that, when separating the mononuclear and polymorphonuclear layer of the blood samples for analysis, both layers showed expression of Tg-mRNA, thereby suggesting that Tg-mRNA transcription also takes place in circulating white blood cells.

The observations regarding the detection of Tg-mRNA in patients and tissues where it should not be detected can be attributed to a phenomenon called ‘illegitimate transcription’ (Chelly *et al*, 1989): any gene is expressed in any cell at very low, but detectable, levels.

### Filtering out illegitimate transcription

Savagner *et al* (2002) tried to circumvent this problem by using prostate-specific antigen (PSA) mRNA to determine the level of illegitimate transcription. Any patient expressing Tg-mRNA in higher quantities than PSA-mRNA was expected to have circulating follicular thyroid cells. Thus, they tried to filter out the non-thyroid expression of Tg-mRNA. Using this method, Savagner *et al* found that serum Tg-mRNA detection has a better sensitivity for detecting recurrent thyroid cancer than serum Tg measurement, especially during LT4 suppression therapy. Instead of PSA, Eszlinger *et al* (2002) and Span *et al* (2003) used beta-actin-mRNA transcription levels for correcting the illegitimate transcription. However, their results showed no statistical differences between patients and controls with respect to corrected Tg-mRNA expression levels.

The results of various studies are summarised in Table 1

**Table 1:** Summary of results of various studies into the usefulness of Tg-mRNA detection

**Study**	**Quantitative?**	**False positive**	**False negative**	**True positive**	**True negative**	**Positive controls**	**Negative controls**
Ditkoff	No	7/78	0/9	9/9	71/78	0/15	15/15
Biscolla	No	3/19	5/14	10/15	16/19	—	—
Ringel (1999)	Yes	13/33	13/74	61/74	20/33	—	—
Savagner	Yes	3/15	6/25	19/25	12/15	—	—
Bojunga, method 1	No	63/137	4/13	9/13	74/137	30/135	105/135
Bojunga, method 2	No	111/137	2/13	11/13	26/137	102/135	33/135
Elisei	Yes	22/29	2/17	15/17	7/29	17/20	3/20
Takano	Yes	All patients and controls are positive					
Bugalho	Semi	No difference in expression levels between individuals with and without thyroid glands					
Bellantone	No	Varies according to histology					
Eszlinger	Yes	No difference between patients with and without metastases or other thyroid diseases					
Span	Yes	No difference between patients and controls					

. For comparison of the different studies, a patient was considered to be positive for the presence of thyroid (cancer) tissue if he/she had proven thyroid cancer, and was showing either detectable serum Tg levels or showed scintigraphic evidence for the presence of disease when blood was drawn for Tg-mRNA detection.

## DISCUSSION

The question we addressed here was whether Tg-mRNA detection in peripheral blood can be used for follow-up in differentiated thyroid cancer.

Thyroglobulin mRNA detection certainly does not turn out to be specific for the presence of metastatic thyroid cancer: Tg-mRNA is detected in peripheral blood samples of patients with benign thyroid disorders and even in samples from healthy subjects (Ringel *et al*, 1998; Tallini *et al*, 1998; Takano *et al*, 2001; Savagner *et al*, 2002; Elisei *et al*, 2004). This suggests that Tg-mRNA-producing cells are present in blood even in patients without thyroid cancer, which could be attributed to illegitimate transcription of Tg-mRNA, or could mean that cell shedding is a physiologic rather than pathologic process, taking place even in normal thyroids.

Especially remarkable is the finding of Bojunga *et al* that changing the number of PCR cycles in analysis completely changes the specificity of Tg-mRNA detection. This suggests that a relatively low level of expression of Tg-mRNA transcription also takes place in many other cells in the body, which, when sufficiently enhanced, will also be detected when analysing with PCR for the presence of circulating thyroid cells. This study certainly provides compelling evidence for the occurrence of illegitimate transcription of Tg-mRNA.

This hypothesis is certainly supported by the finding of Selliti *et al* (2000), who reported that human kidney cells respond to TSH stimulation with the production of Tg-mRNA. Additionally, when using an immunofluorescent staining with a monoclonal anti-Tg antibody, positive staining can be identified in the cytoplasm of mesangial cells.

### Possible enhancements to Tg-mRNA detection

Under the hypothesis that Tg-mRNA is expressed at low levels in various human cell lines, and at higher levels in normal or neoplastic thyroid cells, one could consider using a quantitative RT–PCR reaction with a clearly defined threshold for detecting Tg-mRNA. However, this might not be the solution. No significant differences in expression levels between patients with thyroid cancer and subjects without thyroid disorders or with benign thyroid conditions could be detected by Takano *et al* (2001) and Fenton *et al* (2001).

Determining the level of illegitimate transcription and correcting for it, like Savagner *et al*, Eszlinger *et al* and Span *et al* did, is also still open to further research: using PSA-mRNA should at least be further validated. Accordingly, one can consider investigating mRNA molecules other than PSA-mRNA or beta-actin-mRNA for correction for illegitimate transcription.

Another option is to further experiment with varying sensitivities of the RT–PCR procedure: for example, 30 cycles of PCR instead of 40 when using the same technique as Bojunga *et al*. However, this option is also still open to research.

Ringel (2004) also suggested some interesting possibilities for further research using RT–PCR in the follow-up of thyroid carcinoma.

### Alternatives to Tg-mRNA

As an alternative to the detection of Tg-mRNA, several groups have investigated the possibility of using measurement of mRNA of other thyroid-specific proteins in the follow-up of differentiated thyroid carcinoma. Biscolla *et al* (2000) in their study also investigated the possibility of detecting sodium–iodine symporter (NIS) mRNA. Their results however showed no benefit from these measurements: NIS-mRNA detection was inferior to Tg-mRNA detection. Tallini *et al* (1998) in their study correlated the detection of thyroid peroxidase (TPO) mRNA and Tg-mRNA. Thyroid peroxidase mRNA was detected in the same samples of peripheral blood as Tg-mRNA and was not found in samples in which Tg-mRNA was lacking. Finally, Roddiger *et al* (2002) investigated the use of TPO-mRNA exclusively. Thyroid peroxidase mRNA expression in peripheral blood was detected in significantly more number of patients with thyroid disease than in control patients. It is also correlated with the presence of metastases, and in patients without known metastases it is correlated significantly with grade, lymph node stage at the time of diagnosis and Tg levels. Based on these data, further investigation of TPO-mRNA seems warranted.

Pauws *et al* (2000) have already analysed the entire spectrum of transcripts from thyrocytes, using a method called ‘serial analysis of gene expression’ (SAGE). More on this method can be found at the URL www.sagenet.org. It should be analysed which of these transcripts in the thyrocyte gene profile are unique to thyroid epithelial cells. Any thyroid-specific mRNA transcript would also be a candidate for use in follow-up. Perhaps the next step could even be to analyse each patients' thyroid carcinoma for its specific gene profile and monitor the patient by detecting circulating cells matching the profile at key points. Such key points would have to be determined in future research.

## USING RT–PCR

The RT–PCR for Tg-mRNA has so far mainly been investigated for use in the regular follow-up of all patients with differentiated thyroid carcinoma, using only one measuring point. Even if Tg-mRNA RT–PCR eventually does not turn out to be useful in the regular follow-up of differentiated thyroid cancer, there might be specific subsets of patients in whom this technique could be used. In patients with medullar thyroid carcinoma, nonquantitative RT–PCR for calcitonin mRNA turns out to correlate with the presence, extent and aggressiveness of metastatic disease (Saller *et al*, 2002). In this study by Saller *et al*, it was also found that in patients who had a clinically significant response to chemotherapy, calcitonin mRNA had become undetectable.

Perhaps RT–PCR for Tg-mRNA could be used in an analogous way for the monitoring of response to therapy in patients with known metastatic thyroid cancer, especially in those patients in whom normal Tg measurements are not reliable due to the presence of Tg antibodies. Especially, monitoring the progression of Tg-mRNA expression levels over time might prove useful.

A single positive sample at any level might not be considered irrefutable proof for the persistence or recurrence of disease, due to phenomena such as illegitimate transcription, but a Tg-mRNA level rising over time will reflect increasing activity of normal or neoplastic thyrocytes. The RT–PCR analysis could possibly detect recurring or persisting disease much earlier than Tg measurements could, especially during LT4-suppressive therapy.

## CONCLUSION

Based on the various studies reviewed in this paper, it can be concluded that at this time there are still too many contradicting results on the usefulness of Tg-mRNA detection in peripheral blood; this technique can therefore not yet be considered a proven and reliable method of following patients after treatment for differentiated thyroid carcinoma.

Further research, for example, whether different levels of sensitivity of arrays could produce a useful level of specificity, or the usefulness of other thyroid-specific gene transcripts, such as TPO-mRNA, should however be performed; further thought should also be given to the use of complete gene profiles of the tumour in the follow-up of differentiated thyroid carcinoma.
